# The Effect of Race and Area Deprivation on Symptom Profiles over the Course of Early-Stage Breast Cancer

**DOI:** 10.21203/rs.3.rs-3649299/v1

**Published:** 2023-11-28

**Authors:** Hiba Abujaradeh, Julia O’Brien, Susan R. Mazanec, Catherine M. Bender, Isabelle M. Schlemmer, Adam M. Brufsky, Elham Nasrollahi, Margaret Rosenzweig

**Affiliations:** University of Pittsburgh; University of Pittsburgh; Case Western Reserve University; University of Pittsburgh; University of Pittsburgh; UPMC Hillman Cancer Center; UPMC Hillman Cancer Center; University of Pittsburgh

**Keywords:** racial disparities, breast cancer, symptom burden

## Abstract

**Purpose:**

This study compared common symptoms (fatigue, pain), overall physical functioning and changes over time between Black and White women receiving early-stage breast cancer (ESBC) chemotherapy.

**Methods:**

A longitudinal, repeated measures comparative design was employed. Time points of symptom measurement (PROMIS domains) at baseline, mid and end point were adjusted as per patient chemotherapy schedule.

**Analyses:**

Linear mixed models were applied.

**Results:**

There were 147 patients, 36% Black 64% White (54±12 years) recommended to receive early-stage breast cancer chemotherapy with adequate data for symptom analysis.

**Pain:**

Main effect of race was significant (*F*(1, 390) = 29.43, *p*<.001) for pain with Black patients experiencing significantly higher pain scores compared to White patients at pretherapy (Mean Difference; MD=3.7, p=.034), midpoint (MD=5.8, *p*=.002), and endpoint (MD=7.8, *p*<.001).

**Fatigue:**

Fatigue significantly increased (deteriorated) at endpoint (MD_T1-T3_= 8.7, *p*<.001) for Black patients. Among White patients, fatigue significantly increased at midpoint (MD_T1-T2_= 5.7) and at endpoint (MD_T1-T3_=10.1, *p*<.001; MD_T2-T3_=4.3, *p*= .017). **Physical function:** Black patients had significantly lower physical function scores compared to White patients at midpoint (MD=4.0, *p*=.027). Physical function decreased by endpoint in Black (MD_T1-T3_=7.8, *p*<.001), and White patients (MD_T1-T3_=7.7, *p*<.001).

**Conclusion:**

Symptom burden significantly increased over the course of chemotherapy for all patients. Scores for pain and physical function were higher overall for Black patients and deteriorated at a greater rate for Black vs. White women over the course of chemotherapy. This assessment holds implication for proactive assessment and mitigation strategies.

## INTRODUCTION

Breast cancer is the second most deadly form of cancer among women in the United States and is the leading cause of cancer death among Black women [1, 2]. In recent years, the mortality rate from breast cancer has decreased due to increased emphasis on early detection and more effective treatments [3]. Despite improvements in early detection [4], disparities in treatment and health outcomes remain, with Black American women having the highest mortality rate related to breast cancer among all racial groups [3]. Several factors are associated with breast cancer survival disparity including later disease presentation [4] and earlier age at diagnosis for Black women, a historic distrust of the health care system influencing screening and treatment decisions, and disproportionately poor communities that may offer few supports along the breast cancer care continuum.

Once cancer is diagnosed and treatment begins, disparities exist in the toxicities experienced during treatment. The 2002 State of the Science Conference for Cancer Symptom Management concluded that “all subjects with cancer should have optimal symptom control from diagnosis throughout the course of illness, regardless of personal and cultural characteristics [5].” Fifteen years later, this goal has not been achieved [6, 7]. To move forward in improving survival and health equity for Black women with breast cancer, further research is needed to deduce why Black women suffer disproportionately with symptom burden during chemotherapy [8].

### Racial Disparities in Symptoms Burden

Symptoms burden defined as the “subjective, quantifiable prevalence, frequency, and severity of symptoms placing a physiologic burden on patients and producing multiple negative, physical, and emotional patient responses [8].” Historically, during ESBC, symptom burden increases from baseline to the midpoint of cancer treatment, at which point it remains stable until ESBC is terminated. Worsening symptom burden has been attributed to increasing gastrointestinal symptoms, fatigue, insomnia, and concerns with appearance [9, 10]. Black women experience more severe symptoms during and post treatment for ESBC [11]. Pain and fatigue are the most common distressing symptoms experienced by patients receiving treatment during early-stage cancer treatment. The impact of these symptoms is poor quality of life, and overall diminution of physical function with implications for social, employment and family roles. It is imperative to examine the racial disparities in these symptoms prior to chemotherapy initiation, during and at the completion of chemotherapy.

### Pain

Black women report more severe pain during ESBC than White women [12, 13]. Ongoing disparities in the treatment of Black patients’ pain is a contributing factor, as Black patients are less likely to receive guideline concordant care and are more likely to have their pain underestimated by healthcare providers [14]. Additionally, higher pain intensity is associated with higher pain interference (i.e., the effect of pain on activities: cognitive, social, physical, and recreation), fatigue, sleep disturbances, depression, and anxiety [12, 15].

### Fatigue

Compared to white women with breast cancer, Black women have higher levels of fatigue across domains (physical, emotional, etc.) shortly after diagnosis as well as during the early post-treatment survivorship phase (i.e., 6 to 18 months posttreatment) [16]. Emotional fatigue declined, and vigor increased from diagnosis to approximately 6 months post-treatment in White patients; whereas these symptoms remain stable among Black women with ESBC [16].

#### Physical Function.

Disparity in physical function has been reported between Black & White women during treatment and during cancer survivorship [17, 18]. Black women were more than twice as likely to have a greater degree of physical functioning li mitation compared to white breast cancer survivors, but this finding was significantly attenuated after adjusting for BMI, smoking status, and the presence of other co-morbid conditions associated with physical activity limitations [18]. Another study supported the mediating role of socioeconomic factors where racial differences in physical and functional wellbeing have been 50% attenuated by socioeconomic differences that impact Black women’s ability to manage cancer and treatment-related symptoms such as differences in physical activity and co-morbid conditions [17].

### The Importance of Considering Social Context

Social contextual determinants of health contribute to both the risk and outcomes of breast cancer and may also contribute to disparities in symptom burden. Social contextual factors include race and gender, but also include socially patterned access to social, education, and economic resources, as well as exposure to other environmental hazards within communities. Lifetime cumulative stress caused by the stressors of poverty and discrimination may accelerate aging, and thus the symptom experience [19].

In addition, psychosocial stress in early life has also been associated with elevated inflammation markers among women with breast cancer [20]. Black women are more likely than white women to have lower SES and are more likely to be exposed to psychosocial stressors in early childhood such as abuse and neglect [21]. The cumulative impact of disparities in social contextual factors may increase the risk and adversely impact breast cancer outcomes through allostatic load or more rapid biological aging. The area deprivation index (ADI),a composite of neighborhood characteristics including poverty, housing, employment, and education, is associated with symptom burden, symptoms of depression and anxiety [22], including psychological well-being and quality-of-life [23] in patients with cancer. Little research has looked to area deprivation within longitudinal data.

Previous work exploring racial differences in symptom burden during ESBC has focused primarily on pain [24], and retrospective assessments were often cross—sectional or time points were not individualized for each patient treatment course of therapy [12, 24], which may not reflect a granular, over time assessment of symptoms during the treatment trajectory. This study aims to advance our understanding of racial disparity in symptom burden during ESBC while considering the individual chemotherapy schedule and overcoming these methodological shortcomings.

## PURPOSE

Limited studies have assessed racial disparities in pain, fatigue, and physical function prior to chemotherapy initiation and changes over the course of treatment, while adjusting for individual timing of chemotherapy for BC patients. The aims of this study are to describe and compare the symptom burden (fatigue, pain, and physical functioning) and change over time between Black and White women receiving ESBC chemotherapy while considering social determinants of health.

## METHODS

This study employed a longitudinal, repeated measures (baseline, midpoint, and completion of chemotherapy assessment timepoints were included in this analysis based on patient chemotherapy treatment plan; total timepoints range for all patients = 3–15), comparative, mixed-methods design. Women with early-stage BC in Western Pennsylvania and Northeast Ohio were followed over the course of chemotherapy. A convenience sample of patients who are: female, Black or White race, 18 years of age or older, and prescribed chemotherapy for a diagnosis of invasive breast cancer (stages 1–3) were included. Prior chemotherapy, metastatic breast cancer, impaired cognition, inability to understand English, and receiving treatment outside the clinic of consent were exclusion criteria. Further details regarding recruitment, enrollment, and data collection are reported by Nugent et al [25].

Prior to Covid – 19 research restrictions, eligible participants were approached by a research assistant in the clinic prior to receiving their first chemotherapy treatment. Following Covid-19 restrictions, the recruitment and consent were obtained by telephone and online. Informed consent was obtained and baseline measures were completed by phone, online, or via paper forms. To capture the full range of changes in symptoms distress over the trajectory of ESBC chemotherapy, we included three time points: prior to chemotherapy (i.e., baseline), midpoint of treatment, and completion of chemotherapy (endpoint) for each patient. The data collection schedule was tailored to the individual patient’s chemotherapy schedule. For example, if a patient needed to have a 7-day treatment delay due to neutropenia, the data collection schedule was adjusted by 1 week.

### Ethical approval

was received from the Institutional Review Boards of (1) University of Pittsburgh (IRB number 19050299) and (2) University Hospitals Cleveland Medical Center (IRB number 02-18-60C).

### Measures

#### Area Deprivation.

The **Area Deprivation Index** (ADI) is a multidimensional evaluation of a neighborhood’s socioeconomic status [26]. The Area Deprivation Index (ADI), developed by the Health Resources and Services Administration [26], allows for neighborhood rankings by socioeconomic status deprivation, a composite of several factors (income, education, employment, and housing quality). Scores range from 0 to 100, with higher scores indicating higher deprivation. National ADI was derived by entering participants’ home addresses at the time of study enrollment into a publicly available interactive website [27]. Post office boxes are neither considered geographically representative nor included within American Community Survey metrics; thus, ADI was not calculated for participants who entered P.O. Boxes. The ADI median (61) was used to categorize participant into high ADI (n = 74; 50.7%), meaning living in areas of greater deprivation, and low ADI (n = 72; 49.3%), indicating living in areas of less deprivation.

#### Race.

Patient race was identified as either White or Black based on both self-reported demographic questionnaire and medical chart review.

### Patient-Reported Outcomes Measurement Information System (PROMIS – 29 Profile v 2.0).

The PROMIS-29 Profile (v2.0) was used to measure symptom burden [28]. The PROMIS-29 is 5-point Likert scale and it includes seven domains (anxiety, depression, fatigue, sleep disturbance, physical function, ability to participate in social roles and activities, and pain). For this paper, symptom burden was defined through measurements of fatigue, pain, and physical function. Raw scores for all domains were converted into T-scores using the Health Measures System (mean of general US adult reference population = 50, SD = 10). Higher PROMIS T-scores represent more of the concept being measured (i.e., worse fatigue and pain and better physical function). The clinical threshold to evaluate within-group change or to make a between-group comparison generally ranges between 2 and 6 T-score points [29].

### Analytic Approach

Data were analyzed using IBM^®^ SPSS^®^, version 27 [30]. Descriptive data (mean, standard deviation, frequency, and percentages) for raw data were provided. Pearson’s correlations (Spearman coefficients) were used to examine the bivariate associations of study variables. Linear mixed models were applied to determine whether significant differences existed among PROMIS domains over the three time points between Black and White patients. In the linear mixed model, we adjusted for area deprivation and then examined whether symptoms burden (pain, physical function, and fatigue) varied by race over the three time points.

## RESULTS

### Participants

There were 147 patients, 36% Black, 64% White with a mean age of 54 ± 12 years. Time average between prechemotherapy and midpoint (42 ± 16 days = 5.6 ± 2.3 weeks) and between midpoint and endpoint (71 ± 22days = 9.8 ± 3.2 weeks). Demographic and clinical characteristics of the sample are presented in Table 1.

### Primary Outcomes

Comparative statistics for primary outcomes and other study variables are presented in Table 2.

#### Pain.

Black patients, compared to White patients, experienced significantly higher scores for pain across all time points, with a mean difference (MD) of 3.7 (p = .034) prior (baseline) to chemotherapy initiation, 5.8 at midpoint (*p* = .002), and 7.8 by completion of chemotherapy (*p* < .001). After adjusting for area deprivation, the main effect of race was still significant (F(1, 390) = 29.43, p < .001) for pain with Black patients experiencing significantly higher pain scores compared to White patients at midpoint (Mean Difference; 4.7, *p* = .02). This disparity was more pronounced by the completion of chemotherapy (MD = 7.8, *p* < .001). Pain scores deteriorated significantly over time for Black patients (within group change) who reported significant increase in pain scores by the completion of chemotherapy (MD_T1−T3_=6.2, *p* = .010; MD_T2−T3_=5.8, *p* = .024). There were no significant changes in pain scores over time among White patients. Refer to Table 3 for marginal means contrasts for mixed models depicting within group changes and Table 4 for marginal means contrasts between Black and White at the three time points.

#### Fatigue.

Adjusting for area deprivation, the main fatigue scores significantly increased (deteriorated) by endpoint (MD_T1−T3_= 8.7, p < .001) for Black patients. Among White patients, fatigue significantly increased by midpoint (MD_T1−T2_=5.7, p < .001) and at endpoint (MD_T1−T3_=10.1, p < .001; MD_T2−T3_=4.4, p = .017).

#### Physical function.

Adjusting for area deprivation, main effect of race was significant (F(1, 391) = 11.00, p < .001) for physical function. Black patients had significantly lower physical function scores compared to White patients at midpoint (MD = 4.0, p = .027). Physical function decreased among Black patients by endpoint (MD_T1−T3_=7.8, p < .001), and in Whites by at endpoint (MD_T1−T3_=7.7, p < .001; MD_T2−T3_=4.8, p = .002).

### Associations of area deprivation and study variables

Of all correlations examined between ADI and symptoms burdens and physical function at the three time points, living in areas of greater deprivation was associated with greater pain interference (r=−.227, p = .002, 56.3 vs 50.1 ) and worse physical function (r=−.190, p=−.035, 40.6 vs 42.4) at the completion of chemotherapy.

## DISCUSSION

This paper compared symptom burden (fatigue, pain, and physical function) and the changes experienced throughout chemotherapy between Black and White women with early-stage breast cancer. The importance of this study lies in its novelty of examining these commonly reported symptoms at a time point often overlooked in the literature—prior to chemotherapy initiation. Furthermore, tracking changes at time points that reflect the full symptom trajectory including mid and endpoint of chemotherapy for each patient while considering the influence of area deprivation.

### Notable racial disparities in pain present at pretherapy and escalate over the course of chemotherapy.

Prior to chemotherapy initiation, Black patients reported pain scores 4 points greater than White patients indicating a clinically significant difference. This could linked to the distress occurring while anticipating treatment [31], psychological and physical symptoms, area deprivation, and worsened economic hardships [32]. This disparity continued to escalate over the course of chemotherapy, with Black patients reporting 6 points greater at midpoints and 8 points greater toward the completion of chemotherapy. This aligns with a cross-sectional study of 40 women with BC stages I-III, status post–initial radiation or chemotherapy for breast cancer, where Black patients (58%) had significantly higher pain scores [12] and another study where Black patients reported more pain-related interference with function than White patients [33]. Our study contributes to the existed knowledge of racial disparities in a period that is overlooked in the literature; prior to chemotherapy initiation; and showed that the exacerbation of pain was greater among Black women over the course of chemotherapy.

Furthermore, within group changes emphasized disparity as a clinically notable deterioration in pain scores over the course of chemotherapy was evident for Black patients only. The continuous deterioration in pain scores for Black patients across the chemotherapy aligns with a recent study by Hu et al. where Black women with ESBC, followed from the initiation of chemotherapy to one year later, were more likely to experience significant increases in physical and psychological symptom burden during chemotherapy. This greater symptoms burden was associated with less adherence to adjuvant endocrine therapy suggesting that better symptom management for Black women can reduce racial disparities in adherence and cancer outcomes [34]. Our findings, along with previous research on racial disparities, provide evidence that Black patients endorse higher rates for pain [35]. However, evidence has shown that Black patients receive inadequate and biased pain assessments and treatment by clinicians and other healthcare providers [36]. Measures ensuring that clinicians and other healthcare providers deliver appropriate assessment and pain assessment to all patients should be taken.

### Adjusting for Area deprivation lessened racial disparity in pain prior to chemotherapy initiation.

Area deprivation broadly refers to the lack of economic and social resources in a neighborhood [37]. After we adjusted for area deprivation in the models, the racial differences in pain at baseline were not statistically significant. In addition, patients residing in areas of greater deprivation from this sample, reported higher pain and worse physical function by the completion of chemotherapy. This is consistent with previous studies where patients with advanced cancer who lived in areas of greater deprivation reported worse symptom burden and worse symptoms of depression and anxiety [22]. Clinicians need to consider neighborhood deprivation when examining racial disparity. The ADI can be utilized in healthcare systems as it is an objective and publicly available measure of social context. High ADI scores can prompt healthcare providers to conduct further assessment of the patients socioeconomic needs and provide the appropriate referral and support within the community. This could mitigate the influence of neighborhood deprivation on racial disparities in symptoms burden.

### Racial disparities were noted for physical function at midpoint.

White patients and Black patients experienced deterioration in physical function by the end of chemotherapy, Black patients experienced this deterioration earlier in the chemotherapy course with significantly lower physical function compared to White patients at midpoint. This could be attributed to the worsening in pain levels that Black patients reported compared to their White counterparts. While there are many interventions to target poor physical function such as support groups, exercise, nutrition, and mindfulness programs, the success of these for Black patients during treatment is not identified [38, 39]. Culturally tailored interventions have shown improvement in other areas of breast cancer such as improving screening rates among African American and other minorities [40]. Culturally tailored symptom assessment and management needs to be a top priority to ensure personalized cancer care for not just treatment, but in symptom assessment and management as well.

### Strengths and Limitations

To our knowledge, our study is the first to look the racial differences in the most common reported symptoms of pain and fatigue and physical function while accounting for the influence of area deprivation and considering patient-individualized timing of chemotherapy initiation, midpoint, and completion. This study addressed the methodological limitations identified in previous research including the utilization of longitudinal data with a focus on time points that reflect the full picture of changes in symptoms while receiving chemotherapy, including a substantially larger sample size, and accounting for area deprivation which is considered a valid measure reflecting patient social status and associated with health-related outcomes. However, these findings should be interpreted with caution, considering any distress or anxiety that triggered by the COVID-19 pandemic [41], which might have influenced the symptoms burden.

### Clinical Implications and Future Research

The baseline symptoms that patients bring to BC prior to chemotherapy initiation and the rate at which symptoms deteriorate must inform medical oncology practice. Practitioners and clinicians should consider symptom burden prior to chemotherapy initiation to accurately identify patients with prior symptoms that could be related to other comorbidities or the stage of diagnosis and avoid mistakenly attributing these symptoms as chemotherapy-related toxic effects.

Symptom management strategies need to be patient-centered, incorporating the differences that race may bring to the symptom experience during breast cancer chemotherapy. Frequent reports of physicians not recognizing the severity of symptoms underline the need for a change systemically, as providers are only able to initiate treatment if they are aware of the symptom burden [42].

Black patients have the greatest unmet needs regarding symptom burden but are also less likely to be assertive and communicate concern efficiently with their physicians [43, 44]. While this may primarily be a problem of cultural barriers in a non-concordant patient-physician relationship [45], a standardized measurement of symptom assessment and management is essential to attenuate the likelihood of chemotherapy dose reduction or cessation for Black patients.

Understanding symptoms burden within the context of race prior to and during chemotherapy could provide better understanding of the influence of racial disparities on the breast cancer chemotherapy experience. Establishing these symptom differences can help to provide the impetus to proactive, targeted symptom management.

## Figures and Tables

**Table 1:** Descriptive Statistics for Demographics Characteristics (N = 147)

**Age (years)†**	**52.99 ±12.31 (25–80)**

Race‡	

Black or African American	53 (35.6)

White	96 (64.4)

Ethnicity‡	146 (98)
Non-Hispanic	0 (0)
Hispanic	3 (2.0)
Unknown	

Marital status‡	79 (53.0)
Partnered	70 (47.0)
Non-partnered	

Employment status‡ (n = 246)	

Employed	141 (57.3)

Non-Employed	105 (42.7)

Education‡ (n = 243)	28 (28.8)
High school or less	172 (70.8)
College and more	
Insurance‡	3 (2.0)
No insurance	70 (47.0)
Public	76 (51.0)
Private	114 (76.5)
Religion background/preference‡	35 (23.5)
Yes (Christian, Jewish, Muslim)	4 (2.7)
No	34 (22.8)
Importance of religion/spirituality‡	76 (51.0)
Not important at all Somewhat important Extremely important Not applicable	35 (23.5)
Somewhat important	
Extremely important	
Not applicable	

Pain	49.76 ±10.11

Fatigue	48.32 ±10.10

Physical Function	49.35 ±8.92

**Table 2:** Comparative statistics for Study Variables

	Total	Black	White	
Physical function Time1	49.3(8.9)	47.9 (10.3)	50.2 (8.0)	
	46.0(8.7)	43.3(9.4)	47.4 (8.1)	
	41.6(8.5)	39.8 (9.2)	42.5 (8.0)	
Pain Time 1	49.8(10.1)	52.1 (11.0)	48.5(9.4)	
	49.2(9.3)	53.0 (10.6)	47.2(7.9)	
	52.8(11.5)	58.0 (12.2)	50.2(10.3)	
Fatigue Time 1	48.3(10.1)	49.2(10.8)	47.9(9.7)	
	53.8(10.2)	54.1(11.9)	53.6(9.2)	
	58.0(9.6)	58.1(11.1)	57.9(8.7)	
Comorbidities				
Cardiopulmonary				<.001
Yes		41 (77.4%)	38 (39.6%)	
No		12 (22.6%)	58 (60.4%)	
Other cancer				.033
Yes		1(7.7%)	12 (12.5%)	
No		52(98.1%)	84 (87.5%)	
BMI		34.3(6.8)	29.9(7.7)	<.001

ADI: Area deprivation index;

**Table 3:** The Marginal Means Contrasts for the Mixed Models: Within Group Changes

The Marginal Means Contrasts for each Combination of Within-Subject Variable: *Pain*			
Contrast	Mean Difference	SE	Significance
Race: Black			
T1-T2	−0.356	2.099	1.000
T1-T3	−6.181*	2.083	.010
T2-T3	−5.825*	2.187	.024
Race: White			
T1-T2	1.067	1.509	1.000
T1-T3	−1.733	1.509	.754
T2-T3	−2.800	1.564	.222
The Marginal Means Contrasts for each Combination of Within-Subject Variable: Physical function			
Contrast	Mean Difference	SE	Significance
Race: Black			
T1-T2	4.193	1.826	.067
T1-T3	7.818*	1.812	<.001
T2-T3	3.625	1.911	.176
Race: White			
T1-T2	2.861	1.318	.092
T1-T3	7.652**	1.318	<.001
T2-T3	4.791*	1.366	.002
The Marginal Means Contrasts for each Combination of Within-Subject Variable: *Fatigue*			
Contrast	Mean Difference	SE	Significance
Race: Black			
T1-T2	−4.938	2.123	.062
T1-T3	−8.737*	2.107	<.001
T2-T3	−3.798	2.212	.260
Race: White			
T1-T2 –5.671*	1.526		<.001
T1-T3 –10.059*	1.526		<.001
T2-T3 –4.388*	1.582		.017

Note: T1 :prechemotherapy; T2:midpoint; T3:endpoint, models were adjusted for area deprivation.

* . The mean difference is significant at the .05 level.

b . Adjustment for multiple comparisons: Bonferroni.

**Table 4:** The Marginal Means Contrasts for the Mixed Models between Black and White

The Marginal Means Contrasts between-Subject Variables for the Mixed Model: Pain			
Contrast	Mean Difference	SE	Significance
Prechemotherapy			
Black-White	3.323	1.833	.071
Midpoint			
Black-White	4.746*	2.028	.020
Endpoint			
Black-White	7.770*	1.976	<.001
The Marginal Means Contrasts between-Subject Variable Adjusting for Area Deprivation: Physical			
Function			
Contrast	Mean Difference	SE	Significance
Prechemotherapy			
Black-White	−2.601	1.593	.103
Midpoint			
Black-White	−3.934*	1.773	.027
Endpoint			
Black-White	−2.767	1.726	.110
The Marginal Means Contrasts between-Subject Variable: Fatigue			
Contrast	Mean Difference	SE	Significance
Prechemotherapy			
Black-White	1.679	1.854	.366
Midpoint			
Black-White	0.947	2.052	.645
Endpoint			
Black-White	0.358	1.998	.858

Notes. Models were adjusted for area deprivation.

* . The mean difference is significant at the .05 level.

## Data Availability

The datasets used and/or analyzed during the current study are available from the corresponding author upon reasonable request.
